# Supplementary material for: Exploring the Pivotal Components Influencing the Side Effects Induced by an Analgesic-Antitumor Peptide from Scorpion Venom on Human Voltage-Gated Sodium Channels 1.4 and 1.5 through Computational Simulation
Source: Toxins (Basel). 2022 Dec 31;15(1):33. doi: 10.3390/toxins15010033 (PMC9864070; doi:10.3390/toxins15010033)
Supplement: Supplementary file 1 [file toxins-15-00033-s001.zip › toxins-2104562-supplementary.pdf]

Supplementary Materials: Exploring the Pivotal Components Influencing the Side Effects Induced by an Analgesic-Antitumor Peptide from Scorpion Venom on Human Voltage-Gated Sodium Channels 1.4 and 1.5 through Computational Simulation

Fan Zhao, Liangyi Fang, Qi Wang, Qi Ye, Yanan He, Weizhuo Xu and Yongbo Song

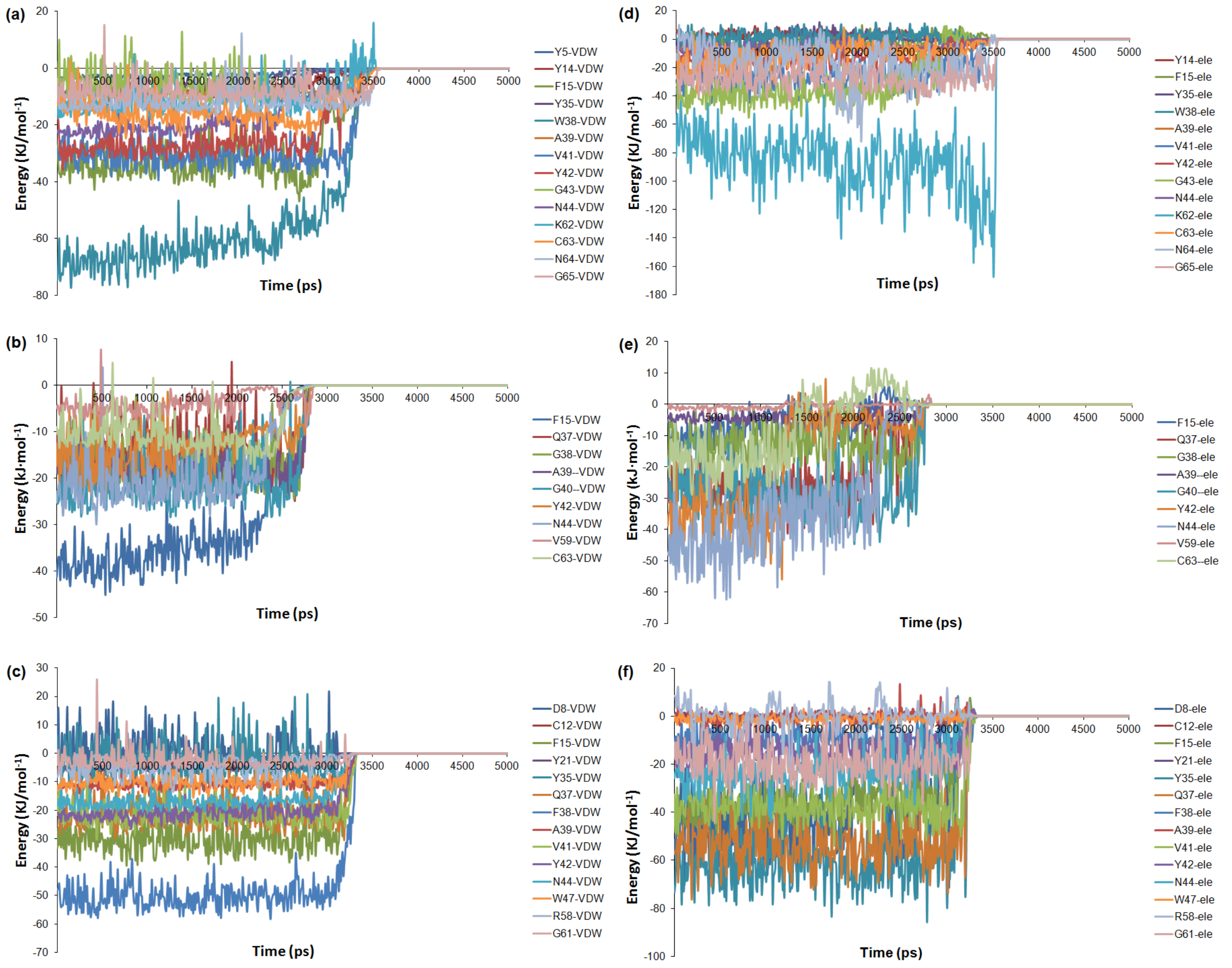

Figure S1. VDW interaction and electrostatic interaction of residues on AGAP and its mutants that directly contact with VSD2s on hNa$_V$1.4. (**a, d**) AGAP; (**b, e**) AGAP^{W38G}; (**c, f**) AGAP^{W38F}. Ele is short for electrostatic interaction.

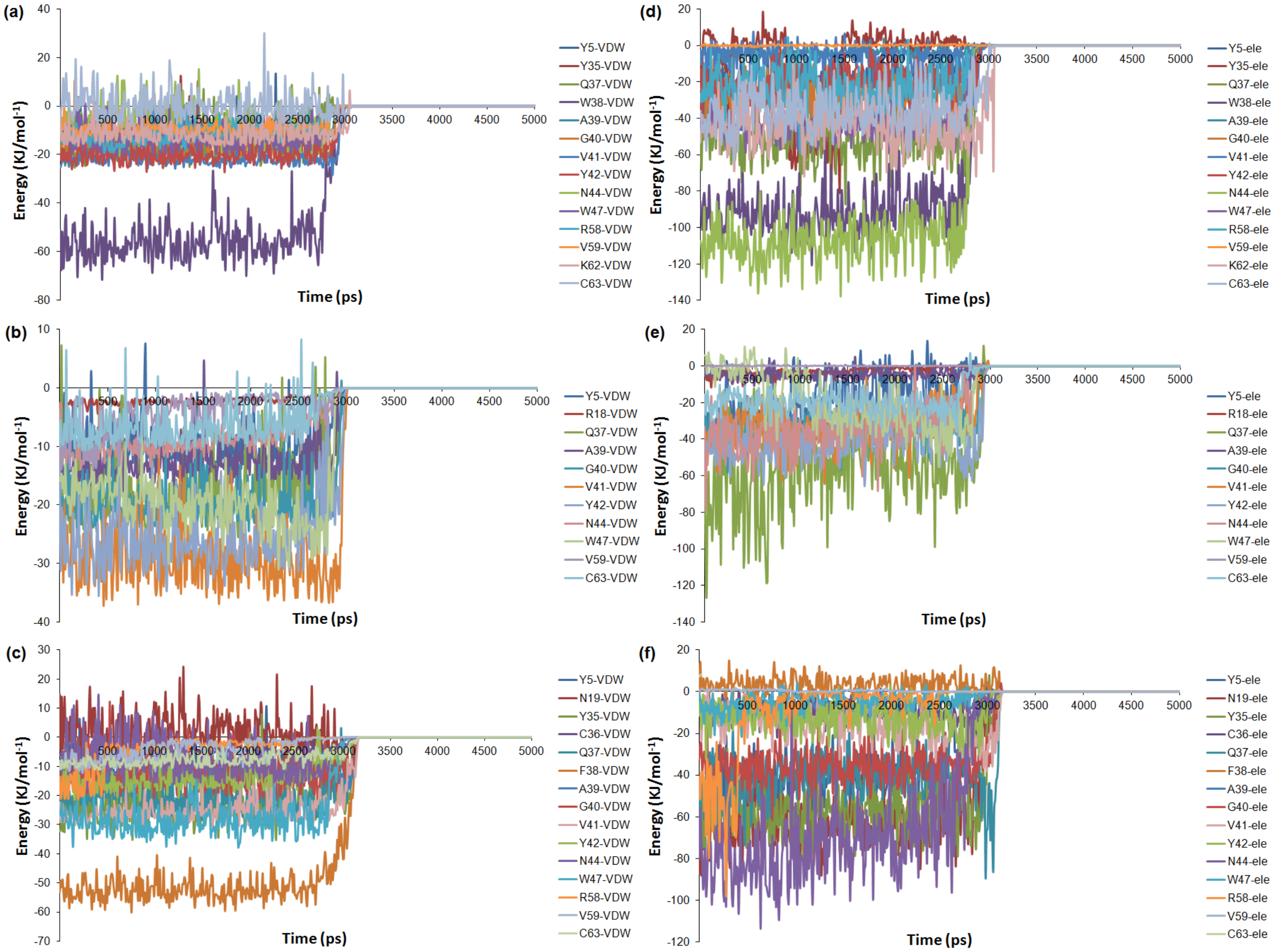

Figure S2. VDW interaction and electrostatic interaction of residues on AGAP and its mutants that directly contact with VSD2s on hNa$_v$1.5. (**a, d**) AGAP; (**b, e**) AGAP^{W38G}; (**c, f**) AGAP^{W38F}. Ele is short for electrostatic interaction.

Table S1. Residues involved in the formations of hydrogen bonds in SMD simulation of AGAP and its mutants with VSD2s on hNav1.4.

Region on AGAP	Residues involed in H-bonding on AGAP	Residues involed in H-bonding on VSD2$^{hNav1.4}$		
		VSD2$^{hNav1.4}$-AGAP	VSD2$^{hNav1.4}$-AGAP^{W38G}	VSD2$^{hNav1.4}$-AGAP^{W38F}
β-sheet I	D8	-	-	Gln105
loop between α-helix and β-sheet I	F15	-	-	Gln105
β-sheet III	Y35	-	-	Asp49
β-turn	Q37	-	Met44	Phe48
	W38	His41	-	-
	G38	-	Tyr42	-
	F38	-	-	-
	G40	-	His41	-
	V41	Met,39, Arg111, Arg114	-	Arg111, Arg114
	Y42	-	Gln105	Arg114
	G43	Glu98	-	-
	N44	Thr53, Glu98	Thr53, Asn56	Glu98
C-terminal	G61	-	-	Arg111
	K62	Glu40	-	-
	C63	Ser108	Gln105	-
	N64	Ser108	-	-
	G65	Leu107	-	-

Table S2. Residues involved in the formations of hydrogen bonds in SMD simulation of AGAP and its mutants with VSD2s on hNav1.5.

Region on AGAP	Residues involed in H-bonding on AGAP	Residues involed in H-bonding on VSD2$^{hNav1.5}$		
		VSD2$^{hNav1.5}$-AGAP	VSD2$^{hNav1.5}$-AGAP^{W38G}	VSD2$^{hNav1.5}$-AGAP^{W38F}
β-sheet I	Y5	Asn43	Asn43	Asn43
loop between α-helix and β-sheet I	N19	-	-	Gln53
β-sheet III	Y35	Glu49	-	Glu50
	C36	-	-	Gln53
β-turn	Q37	Met44, Thr45	Glu40, His41, Asn43	Gln53
	W38	Glu98, Arg111	-	-
	G38	-	-	-
	F38	-	-	-
	G40	His41	His41, Arg111	His41, Arg111
	V41	Asn106, Leu107	Tyr42, Leu110	Tyr42
	Y42	Asn106, Leu107	Asn106	Asn106
	N44	Glu98, Ser102, Met104	Glu98, Ser102	Glu98, Ser102
β-sheet II	W47	Asn43, Thr45	Asn43	-
C-terminal	R58	Asn43	-	Asn43
	K62	Ser108	-	-
	C63	Asn106	Asn106	Asn106

Table S3. Abbreviations in the paper.

Word or Phrase	Abbreviation
voltage-gated sodium channel	VGSC
β-Scorpion Toxin	β-ScTx
analgesic-antitumour peptide	AGAP
four homologous domains formed the α-subunit of VGSC	DI-DIV
voltage sensing domain	VSD
discrete optimized protein energy	DOPE
molecular dynamics	MD
1-palmitoyl-2-oleoyl-sn-glycero-3-phosphocholine	POPC
particle mesh Ewald	PME
root-mean-square deviation	RMSD
steered molecular dynamics	SMD
potential of mean force	PMF
wild type	WT
